# Youth Access to Electronic Cigarettes in an Unrestricted Market: A Cross-Sectional Study from Poland

**DOI:** 10.3390/ijerph15071465

**Published:** 2018-07-11

**Authors:** Lukasz Balwicki, Danielle Smith, Malgorzata Balwicka-Szczyrba, Michal Gawron, Andrzej Sobczak, Maciej L. Goniewicz

**Affiliations:** 1 Department of Public Health and Social Medicine, Medical University of Gdansk, Zwyciestwa 42A, 80-210 Gdansk, Poland; 2 Department of Health Behavior, Roswell Park Comprehensive Cancer Center, Buffalo, NY 14263, USA; Danielle.Smith@roswellpark.org (D.S.); Maciej.Goniewicz@roswellpark.org (M.L.G.); 3 Department of Civil Law, University of Gdansk, 80-309 Gdansk, Poland; m.balwicka@prawo.ug.edu.pl; 4 Department of General and Analytical Chemistry, Division of Laboratory Medicine, School of Pharmacy, Medical University of Silesia, 41-200 Sosnowiec, Poland; mch.gawron@gmail.com (M.G.); asobczak@sum.edu.pl (A.S.); 5 Institute of Occupational Medicine and Environmental Health, 41-200 Sosnowiec, Poland

**Keywords:** e-cigarette access, e-cigarette use, youth, health behavior

## Abstract

*Background*: Electronic cigarette (e-cigarette) use among youths in Poland has become very popular. The aim of this study was to identify the potential points of access to these products by students aged 16–17 years old before implementation of sales restrictions to minors in Poland in November 2016. *Methods*: A school-based, cross-sectional survey was administered in 2015–2016 in 21 secondary/technical schools across two regions of Poland. Analyses focused on 341 students aged 16–17 years old who reported their past 30-day use of e-cigarettes. Pearson Chi-square analyses were utilized to examine the associations between access-related items, e-cigarette use and demographics. *Results*: Among youth e-cigarette users, the most common access to their first e-cigarette was from a friend (38%), followed by purchasing from vape shops (26%). Similar patterns emerged when the students were asked about their access to the currently used e-cigarette. Most youths reported no difficulty in purchasing cartridges/e-liquid containing nicotine (90%). The majority of users (52%) reported buying such products in vape shops. *Conclusions*: Prior to implementing age-related sales restrictions, youth access to e-cigarettes and paraphernalia did not pose any significant barriers. Poland’s introduction of a new age limit on e-cigarette sales may help to limit the number of youths who purchase e-cigarettes from vape shops.

## 1. Introduction

Several epidemiological, clinical and laboratory studies show that although electronic cigarettes (e-cigarettes) are promising harm reduction tools, they also may pose risks to non-smokers who start to use them. According to the U.S. Surgeon General, nicotine should not be used by youths as youths are disproportionately vulnerable to the adverse effects related to nicotine exposure (such as addiction, greater likelihood of using other addictive substances and greater problems with mood, attention and cognition) [1]. Irrespective of nicotine, other ingredients in e-cigarette liquids and aerosols may also pose health risks to users. For example, formaldehyde and acetaldehyde are recognized carcinogens that have been found in aerosols produced in e-cigarettes [2]. Additionally, the flavorings found in e-liquids are appealing to youth users [3] and can present potential respiratory health risks as the effects of repeated inhalation of food-grade flavorings is currently unknown. For instance, benzaldehyde (commonly found in cherry-flavored e-cigarette products) is a respiratory irritant [4], while diacetyl (present in many fruit-flavored e-cigarettes) can cause a condition known as “popcorn lung disease” [5].

Despite these potential health risks, e-cigarette use has become popular among youths [6]. Epidemiological studies show differences in the prevalence of e-cigarette use in this group across countries. The past 30-day e-cigarette use among youths in the U.K. was 2.0% [7], 3.1% in USA [8] and 3.2% in Ireland [9]. By contrast, there are countries where e-cigarettes are used more frequently. Recent prevalence estimates from South Korea suggest that 4.7% of youths report using e-cigarettes in the past 30 days [10]. The rates of youth e-cigarette use are much higher in New Zealand (19.9%) [11]. Our studies on e-cigarette use among youths in Poland have shown significant increases in the use of e-cigarettes from 6.0% in 2011 [12] to 29.9% in 2014 [13].

Researchers have shown that factors associated with e-cigarette uptake among students are similar to those for cigarette smoking. For example, evidence suggests that “curiosity” and “experimentation” serve as factors influencing e-cigarette trial [14,15]. Concerns have been raised that e-cigarette experimentation leads to regular e-cigarette use and may serve as a gateway product for cigarette smoking among youths who may not have otherwise been introduced to nicotine-containing products [16].

Unrestricted access to tobacco products serves as a key factor in initiating nicotine use [17] although little is known about how young e-cigarettes users access and purchase e-cigarette products. It is important to identify and enforce effective strategies that may limit youth access to e-cigarettes as any preventive measures limiting youth access to e-cigarettes need to be balanced with controlled access by adult smokers. In Poland, access to tobacco products, including conventional cigarettes, is limited to those above age 18. Prior to 2016, no parallel age limits were implemented for e-cigarette purchases. The article outlines how youths in Poland accessed e-cigarettes in an unregulated market without age-related restrictions on e-cigarette purchases.

## 2. Materials and Methods

### 2.1. Participants

Data were obtained from a cross-sectional survey performed by the authors in 2015–2016. Detailed methodology was described previously [13,18]. Briefly, data were collected using an anonymous, self-directed paper and pencil questionnaire administered by school teachers to students in 21 secondary and technical schools throughout two regions of Poland (10 in Slaskie and 11 in Pomorskie Voivodeship) using a three-staged stratified cluster sampling design. In total, 2222 students responded to the survey, including 1059 students from schools located in urban areas and 1139 students from schools located in rural areas.

We restricted the scope of our analysis to participants aged 16–17 years old who answered all key demographic questions to evaluate how those under the legal age of purchasing tobacco products accessed e-cigarettes and if they reported any restrictions or problems with purchasing devices. Our analyses focused on youths who reported any use of e-cigarettes in the past 30 days. The analytical sample included students who only used e-cigarettes (exclusive e-cigarette users) and students who reported their past 30-day use of e-cigarettes and smoking tobacco cigarettes (dual users). After exclusions, 341 students remained in the final analytic sample.

### 2.2. Measures

Tobacco and e-cigarette use status was determined by students’ self-reported use of e-cigarettes (exclusive e-cigarette users) or concurrent users of tobacco cigarettes and e-cigarettes (dual users) within the past 30 days. The main outcome measures included responses to the following questions: “Did you have difficulty obtaining an e-cigarette?” (Yes, no); “Where did you get your first e-cigarette?” (Internet, shopping mall, vape shop, received it as a gift, bought on black market, from a friend, other); “Where did you get the e-cigarette you currently use?” (Internet, shopping mall, vape shop, use device that I received as a gift, bought on black market, I borrow an e-cigarette but do not own one); “Have you had trouble getting cartridges/liquid with nicotine?” (Yes, no); and “Where do you usually buy your cartridges/e-liquid? (Internet, shopping mall, vape shop, other)”.

### 2.3. Statistical Analysis

Descriptive statistics were generated for each variable of interest, before Pearson Chi-square analyses were utilized to examine the associations between access-related items, e-cigarette use and demographics. In this present study, *p*-values < 0.05 were considered to be statistically significant. All analyses were conducted in IBM SPSS Version 21.0 (IBM Corporation, Armonk, NY, USA).

## 3. Results

Table 1 displays the demographic information for the sample. Among all e-cigarette users, 33% were exclusive past 30-day e-cigarette users, while 67% were dual users of e-cigarettes and tobacco cigarettes. Largely, there were no statistically significant differences in the demographics of exclusive e-cigarette users and dual users, with the exception of the differences by gender (χ^2^ = 10.725, *p* < 0.001) as more male students reported exclusive use of e-cigarettes. By contrast, numerous differences emerged in product use patterns and characteristics of products chosen by exclusive and dual users. Exclusive e-cigarette users were more likely to use e-cigarettes less frequently, while dual users were more likely to report using nicotine-containing e-cigarettes (Table 1).

**Figure 1:**
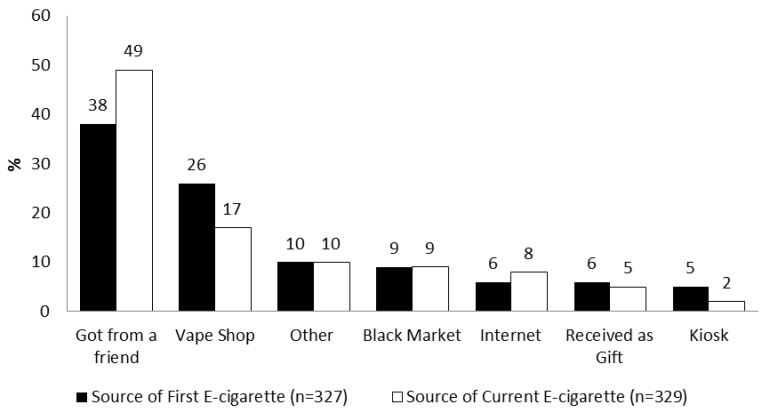
Sources of acquisition for first e-cigarette and current e-cigarette used.

Figure 1 shows the most commonly reported sources of e-cigarette acquisition. The most frequently reported sources of first e-cigarette and currently used e-cigarette were from friends (38% and 49%, respectively). A greater proportion of female students reported obtaining their first e-cigarette from friends compared to male students (42% vs. 35%, χ^2^ = 18.47, *p =* 0.005). A greater proportion of females compared to males reported getting the device they currently use from friends (53% vs. 44%, χ^2^ = 14.60, *p =* 0.024). Vape shops also emerged as a significant point of access to e-cigarettes among youths as 26% of the respondents reported purchasing their first e-cigarette at a vape shop, while 17% reported obtaining the device they currently used from a vape shop. Males were significantly more likely to obtain their first device from a vape shop compared to females (31% vs. 21%, χ^2^ = 18.47, *p =* 0.005) as well as their current device (14% vs. 21%, χ^2^ = 14.60, *p =* 0.024) Dual users were also more likely to obtain their first e-cigarette from a vape shop compared to exclusive e-cigarette users (30% vs. 18%), while exclusive e-cigarette users were more likely than dual users to report obtaining an e-cigarette from friends (53% vs. 31%, χ^2^ = 15.63, *p =* 0.016). Youths in rural areas more frequently reported obtaining their first e-cigarette from the internet compared to youths in urban areas, while they less frequently reported purchasing these e-cigarettes on the black market compared to urban youths (χ^2^ = 12.721, *p =* 0.048). Rural youths were significantly less likely to report purchasing their currently used e-cigarette from a kiosk in contrast to urban youths (χ^2^ = 13.904, *p =* 0.031).

Among all past 30-day e-cigarette users, 52% reported obtaining cartridges/e-liquid from a vape shop, 29% from “other” sources, 14% from kiosks and 6% from online shops. The differences in the source of cartridges/e-liquid acquisition were not statistically significant (χ^2^ = 2.686, *p =* 0.443). One in ten students reported having trouble obtaining an e-cigarette, while 9% reported having trouble obtaining cartridges or e-liquid that contained nicotine. The only significant differences in reported difficulty in obtaining an e-cigarette were by age, with 14% of 16-year-old respondents reporting having difficulty compared to 7% of those aged 17 years old (χ^2^ = 4.673, *p =* 0.31).

## 4. Discussion

According to our knowledge, this is the first study to assess points of access and retail outlets where youths obtained e-cigarettes in an unrestricted market. Our data showed that youths mainly received e-cigarettes from friends, while the second most common retail outlet for purchasing devices was vape shops. Similarly, youths frequently reported buying cartridges/e-liquids in vape shops and in kiosks. The Internet was not a popular way to buy e-cigarette devices or e-liquids. The findings from previous studies suggest that web retailers easily supply underage users [19] due to the absence of effective age-verification measures [20]. Our study challenges the assumption that the most common access to e-cigarettes among youths is via Internet. However, in the countries with more restrictions on the retail sale of tobacco products, youths may be more likely to attempt purchasing e-cigarettes from online stores.

In our sample, the teenagers got their devices mostly from friends, which is consistent with other data from the U.S. showing the association between friends’ use of e-cigarettes with an individual’s e-cigarette use [21]. Consistent with this finding, it seems that Polish youths share their devices often, particularly young women, who were also more likely to be exclusive e-cigarette users. Peer groups influence experimentation with substance use, many of whom supply other youths with e-cigarette devices. Identifying problematic interrelationships among youths that may lead to an increased likelihood of substance experimentation and trial is warranted with respect to growing e-cigarette use experimentation and use [22].

At the time that this study was conducted, there were no age-related sales restrictions in Poland that limited the ability for teenagers to purchase e-cigarettes or nicotine-containing e-liquids from different sources. Similar to New Zealand, Poland had unrestricted advertising and promotion of e-cigarettes until 2016. This needs to be taken into consideration when interpreting the results of our study. As is the case with many other nations, Poland regulates tobacco sales, use and marketing [23]. While representatives of the e-cigarette industry declared that self-regulation measures were sufficient to forbid youth access to e-cigarettes, our study suggests that the voluntary actions taken by retailers may have only marginal effects at best. Enacted in late 2016, the new amendment to the tobacco control law (The Act of 9 November 1995 on Protection of Public Health Against the Effects of Tobacco Use) is in accordance with the European Union Tobacco Product Directive implementation, which requires a ban on sales of these products to people under the age of 18. Even today, based on our knowledge, retailers and internet vendors in Poland are not subject to surveillance, which limits the ability to ensure compliance with the law. To minimize access to e-cigarettes by youths, the Internet sale of these products needs to be closely monitored and online shops should implement strict age verification processes. Retail outlets should be required to verify the age during the purchase of e-cigarettes [24].

This study was subject to limitations. First, there was no objective validation of e-cigarette and combustible cigarette use, such as exhaled CO or measurements of cotinine. Furthermore, our results cannot be generalized to all Polish teenagers as we only researched two regions in Poland. These data were based on self-reports and questionnaires were administered by school teachers, which may be prone to response and reporting bias. However, the likelihood of such bias was assumed to be minimal, since the survey was conducted so that the responders remained anonymous. Finally, our data did not permit us to compare access to tobacco cigarettes and e-cigarettes, especially given the small group of other tobacco product users.

## 5. Conclusions

Our data suggest that prior to implementing age-related sales restrictions, youth access to e-cigarettes and paraphernalia was not restricted by any effective preventive measures. The implementation and future evaluation of Poland’s new policy will provide additional information to support the effectiveness of age-related policy interventions as applicable to e-cigarette use among youths. Future surveillance efforts are needed to assess changes in self-reported access to e-cigarettes. Further research should address the access to e-cigarettes from peers and friends.

## Figures and Tables

**Table 1:** Participant demographics, e-cigarette use patterns, and e-cigarette product characteristics *.

Characteristic	Response	Current E-Cigarette Use				
		Overall (*n* = 341)	Exclusive E-Cigarette Users (*n* = 112)	Dual Users of E-Cigarettes and Tobacco Cigarettes (*n* = 229)	χ^2^ (Excl. vs. Dual)	*p*-Value
Age	16	126 (37%)	44 (39%)	82 (36%)	0.391	0.532
	17	215 (63%)	68 (61%)	147 (64%)		
Sex	Female	165 (48%)	40 (36%)	125 (55%)	10.725	<0.001
	Male	176 (52%)	72 (64%)	104 (45%)		
Place of Residence	Urban	166 (49%)	51 (46%)	115 (50%)	0.660	0.417
	Rural	175 (51%)	61 (55%)	114 (50%)		
Type of School Attended	Secondary School	129 (38%)	49 (44%)	80 (35%)	2.485	0.115
	Technical/Vocational School	212 (62%)	63 (56%)	149 (65%)		
Ever smoked one cigarette	Yes	311 (91%)	82 (73%)	229 (100%)	67.256	<0.001
	No	30 (9%)	30 (27%)	0 (0%)		
Frequency of E-cigarette Use	Everyday	110 (33%)	27 (25%)	83 (37%)	5.466	0.065
	At least weekly	81 (24%)	27 (25%)	54 (24%)		
	Less than weekly	146 (43%)	56 (51%)	90 (40%)		
Puffs per day on EC	<5 times/day	186 (58%)	67 (64%)	119 (54%)	7.305	0.026
	5–10 times	40 (12%)	16 (15%)	24 (11%)		
	10+ times	97 (30%)	21 (20%)	76 (35%)		
Use nicotine in EC?	Yes	276 (87%)	82 (79%)	194 (90%)	7.792	0.005
	No	43 (14%)	22 (21%)	21 (10%)		
Use flavor in EC?	Yes	305 (97%)	105 (98%)	200 (97%)	0.580	0.446
	No	9 (3%)	2 (2%)	7 (3%)		

* Data are presented as *n* (%) for valid responses only. Stratified figures may not sum to the total sample size due to missing response data.
